# Topological quantum criticality in non-Hermitian extended Kitaev chain

**DOI:** 10.1038/s41598-022-11126-7

**Published:** 2022-04-28

**Authors:** S Rahul, Sujit Sarkar

**Affiliations:** 1 Department of Theoretical Sciences, Poornaprajna Institute of Scientific Research, 4, Sadashivanagar, Bangalore, 560080 India; 2 Department of Theoretical Sciences, Poornaprajna Institute of Scientific Research, Bidalur Post, Devanhalli, Bangalore Rural, 562110 India; 3 Graduate Studies, Manipal Academy of Higher Education, Madhava Nagar, Manipal, 576104 India

**Keywords:** Phase transitions and critical phenomena, Topological insulators

## Abstract

An attempt is made to study the quantum criticality in non-Hermitian system with topological characterization. We use the zero mode solutions to characterize the topological phases and, criticality and also to construct the phase diagram. The Hermitian counterpart of the model Hamiltonian possess quite a few interesting features such as Majorana zero modes (MZMs) at criticality, unique topological phase transition on the critical line and hence these unique features are of an interest to study in the non-Hermitian case also. We observe a unique behavior of critical lines in presence of non-Hermiticity. We study the topological phase transitions in the non-Hermitian case using parametric curves which also reveal the gap closing point through exceptional points. We study bulk and edge properties of the system where at the edge, the stability dependence behavior of MZMs at criticality is studied and at the bulk we study the effect of non-Hermiticity on the topological phases by investigating the behavior of the critical lines. The study of non-Hermiticity on the critical lines revels the rate of receding of the topological phases with respect to the increase in the value of non-Hermiticity. This work gives a new perspective on topological quantum criticality in non-Hermitian quantum system.

## Introduction

From the basic axiom of the quantum physics, we know that observables become self-adjoint operators which are represented by Hermitian matrices^1,2^. These systems have real energy eigenvalues due to their Hermitian properties for example the Schr$\ddot{o}$dinger equation. In general the real physical systems interact with environment depending on the degree of interaction which results in a more complex description^3^. The Effective non-Hermitian (NH) provide an intuitive approach along with wide range of applications. The applications emerge from classical settings such as, optical, electrical systems with effective NH Schr$\ddot{o}$dinger equation to the quantum settings^4,5^.

The concepts of non-Hermitian physics found its place in topological states of matter in recent times^4,6^. The introduction of non-Hermiticity altered the concepts of topological systems greatly. The excitation spectrum is no longer real, the definitions of gap in the energy dispersion and the Brillouin zone are completely different when compared to its definitions in Hermitian setting. Features that emerge as a consequence of introduction of non-Hermiticity to the topological systems are complex energy dispersion, EP and modified bulk boundary correspondence^5,7–9^. Complex energy dispersion shows that gap closing and and also the concept of gap is different in non-Hermitian systems. To observe the gap closing behavior the absolute value of the energy spectrum is considered otherwise observing only the real part or imaginary part to identify the topological phase transition will be nonphysical.

An important concept for classification of topological systems is the topological invariant number^10^. For Hermitian systems it is discussed in the above subsections. For non-Hermitian systems, since the curvature function is complex in nature and also the concept of periodic Brillouin zone boundary is altered^11–13^. Hence the regular definition of topological invariant number is not enough in the non-Hermitian setting.

The study of Non-Hermitian systems have gained an immense attention and importance in the recent times when it entered the area of topological systems^6,14–16^ but the criticality in non-Hermitian systems is as interesting as compared to its Hermitian counterpart because of the presence of EP^17,18^. These are the points at which the complex eigenvalues become degenerate and also since it is complex in nature, they come in pairs. The interesting properties of the EP are widely observed in experiments . For the topological systems, the EP have been classified as rings and surfaces^18–21^.

Non-Hermitian system offers a look into a more realistic and interactive setup, in other words, the non-Hermiticity is the interaction of environment with the system or vice versa. *PT* symmetric Hamiltonians are non-Hermitian systems, where these Hamiltonians possess real eigenvalues in the *PT* unbroken phase^4,6,20,22,23^. These *PT* symmetric Hamiltonians are widely used in the field of optics because, controlled dissipation can be achieved in these systems^9,24–30^.

There have been studies on the non-Hermitian Kitaev system hosting MZMs^31–33^. Topological properties and topological invariant number of non-Hermitian system^11,34–38^ are studied where they show stable topological phases and fractional topological invariant number. Many of such non-Hermitian systems show the topological nature and fall under different symmetry classes^39^. There have been tremendous advances in the experimental realizations of non-Hermitian systems in the field of laser physics, acoustics and in the quantum computation^40–45^.

## Motivation

The definitive clarity of physics for Kitaev chain^46^ in presence of non-Hermiticity is understood but the extended Kitaev model (Hermitian) hosts more than one topological phases which is in a way responsible for the appearance of MZMs at criticality. It consists of non-high symmetry critical line where the curvature function diverges at different k values whereas in all other critical lines the excitation the curvature function diverges onnly at a specific k value.

It also possesses multicritical point in which one of them is responsible for the topological phase transition on the critical line to occur. But when it comes to non-Hermitian systems, the excitation spectrum is complex and as a consequence the criticality also changes. Gap closing points are associated with the appearance of EP at which the eigenstates coalesce. Exploration of EP and the effect of $\gamma$ on the EP are important aspects of non-Hermitian systems. The questions on the topological properties like, how does the concept of criticality change? Since it is complex in nature, how does real and imaginary components behave? Does robustness of the MZMs change with respect to $\gamma$? With these important questions, we attempt to answer it by investigating the effects of non-Hermiticity on the extended Kitaev model and its topological phases and criticality.

## Model Hamiltonian

We consider an extended Kitaev chain in presence of non-Hermiticity as follows,

1 $$\begin{aligned} H= & {} - \lambda _1 \sum _{i=1}^{N-1} (c_{i}^{\dagger }c_{i+1} + c_{i}^{\dagger }c_{i+1}^{\dagger } + h.c) - \lambda _2 \sum _{i=1}^{N-1} ( c_{i-1}^{\dagger }c_{i+1} \nonumber \\&+ c_{i+1} c_{i-1} + h.c) -(\mu +i \gamma ) \sum _{i=1}^{N} (1 - 2 c_{i}^{\dagger }c_{i}). \end{aligned}$$

Where $\lambda _1$, $\lambda _2$, $\mu$ and $\gamma$ corresponds to nearest, next nearest neighbor coupling, chemical potential and non-Hermitian factor respectively.

The Hermitian counterpart of this Hamiltonian has been studied explicitly in the references^47–51^ which holds many interesting results such as MZMs at criticality, topological phase transition on the critical line, unique properties of the multicritical points. At the excitation spectra, $E_k=0$, signaling quantum phase transition there exist three such points of this model in the momentum space are at, $k=0$, $k=\pm \pi$ and $k=\cos ^{-1}(-\frac{\lambda _1}{2\lambda _2})$ giving rise to three critical lines $\lambda _2=\mu -\lambda _1$, $\lambda _2=\mu +\lambda _1$ and $\lambda _2=-\mu$ respectively.

After the Fourier transformation, the Hamiltonian (Eq. 1) becomes,

2 $$\begin{aligned} H = \sum _{k} (2(\mu +i \gamma ) - 2 \lambda _1 \cos k - 2 \lambda _2 \cos 2k) c_{k}^{\dagger } c_{k} + i \sum _{k} (2 \lambda _1 \sin k c_{k}^{\dagger }c_{-k}^{\dagger } + 2 \lambda _2 \sin 2k c_{k}^{\dagger }c_{-k}^{\dagger } + h.c). \end{aligned}$$

The Hamiltonian written in terms of Anderson pseudo spin form is given by^49,52^,

3 $$\begin{aligned} H_k = \chi _{z} (k) \sigma _z - \chi _{y} (k) \sigma _y = \left( \begin{matrix} \chi _{z} (k) &{}&{} i\chi _{y} (k)\\ -i\chi _{y} (k) &{}&{} -\chi _{z} (k)\\ \end{matrix} \right) , \end{aligned}$$

where $\chi _{z} (k) = -2 \lambda _1 \cos k - 2 \lambda _2 \cos 2k + 2(\mu + i \gamma ),$ and $\chi _{y} (k) = 2 \lambda _1 \sin k + 2 \lambda _2 \sin 2k$ are the components of the Hamiltonian.

Energy dispersion relation is,

4 $$\begin{aligned} E_k=\pm \sqrt{(\chi _{z} (k))^2 + (\chi _{y} (k))^2}. \end{aligned}$$

Generally for the Hermitian case, the topological invariant number *W* is calculated with the components of Hamiltonian $\chi _{z} (k)$ and $\chi _{y} (k)$, i.e.,

5 $$\begin{aligned} W = \frac{1}{2\pi }\oint \frac{\partial \theta }{\partial k} dk ,\end{aligned}$$

where $\theta = \arctan \left( \frac{\chi _{y} (k)}{\chi _{z} (k)}\right)$. For the non-Hermitian systems, due to the complex nature of the components of the Hamiltonian the integral definition changes^37^.

The energy eigenvalues are complex throughout the parameter space and does not possess any real eigenvalue unlike the *PT* symmetric Hamiltonians^6,53^. Calculating the critical lines becomes tedious for the model Hamiltonian and hence ZMS are used to characterize the topological phases and criticalities.

## Results

### Zero mode analysis for topological characterization

**Figure 1:**
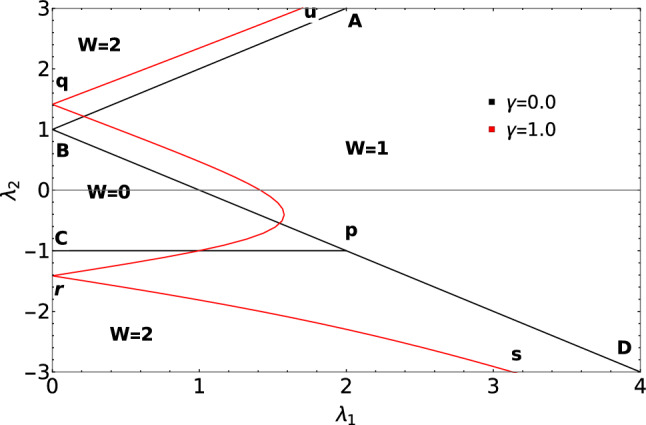
Phase diagram of the model Hamiltonian in presence of $\gamma$ $(=1.0)$. Solid black lines corresponds to the topological phase diagram for $\gamma =0.0$ (Hermitian case).

The introduction of the non-Hermitian factor to the extended Kitaev chain has a significant impact on criticality and topological phase transition. Although all the topological phases remain robust depending on the strength of the non-Hermiticity, a major effect on the criticality is observed. Before we begin the discussion on the effect of $\gamma$ on the criticality and topological phases, we first brief about the criticality and topological phases in the Hermitian case by understanding the phase diagram. Phase diagram of the Hermitian case is also presented in the Fig. 1 for the sake of comparison with the non-Hermitian case. The Hermitian model consists of three critical lines (solid black line) “AB”, “BD” and “CP” distinguishing topological phases $W=0$, $W=1$ and $W=2$.

For the Hermitian case, the topological phases are characterized using integral definition of winding number *W*^47,49,50^ whereas for the non-Hermitian model the topological phases are using ZMS. From the Fig. 1, it can be clearly observed that the critical line “CP” is not present for the non-Hermitian case (critical lines presented in red color).

In the positive $\lambda _2$ region of phase diagram Fig. 1, the effect of $\gamma$ on these critical lines is just a parallel shift. In the negative $\lambda _2$ region, the critical line follows the curved path (q to r) as shown in the Fig. 1. The critical line (r to s) also follows a curved path. These critical lines following the curved path for the non-Hermitian case is the consequence of the non-Hermiticity.

Here construct the phase diagram of the non-Hermitian model using the ZMS.

Topological phases for the non-Hermitian case are characterized using ZMS which is presented as follows. Substituting the exponential forms of $\cos k$ and $\sin k$, Eq. (3) becomes,

6 $$\begin{aligned} H= & {} \left[ 2 \lambda _1 \frac{1}{2} (e^{-ik} + e^{ik}) + 2 \lambda _2 \frac{1}{2} (e^{-2ik} + e^{2ik} + 2 (\mu +i \gamma ))\right] \sigma _z\nonumber \\&+ i \left[ 2 \lambda _1 \frac{1}{2} (e^{ik} - e^{-ik}) + 2 \lambda _2 \frac{1}{2}(e^{2ik} - e^{-2ik}) \right] \sigma _y. \end{aligned}$$

Solving the Eq. (6) for $H^2 = 0$, we obtain the ZMS as,

7 $$\begin{aligned} X_{\pm } = \frac{-\lambda _1 \pm \sqrt{\lambda _1^2 + 4 \lambda _2 (\mu +i \gamma )}}{2 \lambda _2}. \end{aligned}$$

The detailed derivation of ZMS from the model Hamiltonian is relegated in the "Method" Section.

These ZMS (Eq. 7) are nothing but the zeros of a model Hamiltonian that can be written in the form of a complex function^47,54^. Zeros lying inside the unit circle presented in the Fig. 3 indicates topological phase, and zeros lying outside the unit circle represents a non-topological phase whereas zeros lying on the unit circle corresponds to criticality.

In this study, the ZMS are studied as a function of $\lambda _1$ to understand the topological phases and phase boundaries. The absolute value of ZMS is considered because the roots are complex in nature.

The ZMS $X_{\pm } >1$ corresponds to topologically trivial phase whereas $X_{+} >1$ and $X_{+} <1$ corresponds to the topological phase with winding number $W=1$. Correspondingly $X_{\pm } < 1$ represents topological phase with winding number $W=2$^46^.

**Figure 2:**
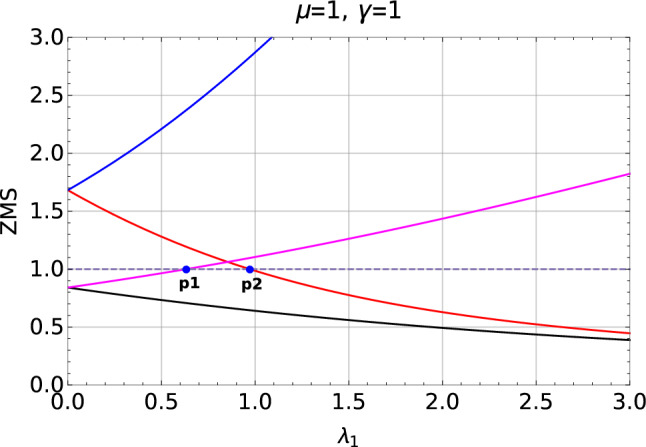
Zero mode solutions plotted with respect to the parameter $\lambda _1$ shows both $W = 0$ to $W = 1$ $(\lambda _2 = 0.5)$ and $W=1$ to $W=2$ $(\lambda _2 = 2.0)$ topological phase transitions. Blue dots (p1 and p2) represent the transition points. The ZMS, $X_{+}$ (red) and $X_{-}$ (blue) are plotted in y-axis.

In the Fig. 2, red and blue curves are the roots representing $W=0$ to $W=1$ transition and magenta and black curves are the roots representing $W=1$ to $W=2$ transition. To specify the transition point using ZMS, parallel to the $\lambda _1$ axis, a reference line (unit line), $y=1$ is drawn which acts in a same way as that of the unit circle drawn to analyze the zeros of a complex function. The point of the intersection between one of the ZMS and the unit line marks the transition point which is represented in blue dots (*p*1 and *p*2) in the Fig. 2.

Value of the roots greater than 1 corresponds to the non-topological phase whereas less than 1 corresponds to topological phase. Transition points *p*1 and *p*2 marks the transitions between $W=2$ to 1 and $W=0$ to 1 respectively with fixed positive value of $\lambda _2$ ($\lambda _2$ = 0.5, 2.0). Keeping track of these transition points via the ZMS method provides the phase diagram of the model Hamiltonian (Fig. 1). The transitions through the curved critical lines in the negative $\lambda _2$ region are analyzed in the further sections.

**Figure 3:**
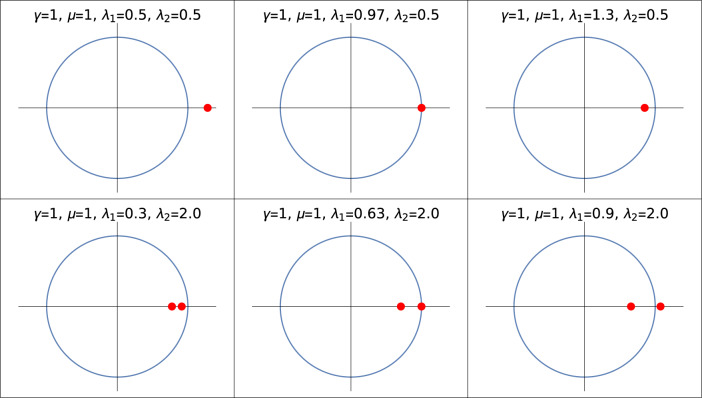
Zeros of a Hamiltonian plotted with respect to a unit circle for the transitions, $W = 0$ to $W = 1$ $(\lambda _2 = 0.5)$ (upper panel) and $W=1$ to $W=2$ $(\lambda _2 = 2.0)$ (lower panel). Two red dots corresponds to the zeros.

In the Fig. 3 we can see the ZMS plotted with respected to a unit circle whereas the unit circle is made into a unit line in the Fig. 2. The zeros falling inside the unit circle corresponds to the topologically non-trivial phase whereas the zeros falling on the unit circle and outside the unit circle corresponds to the transition and topologically trivial phase respectively. In both Figs. 2 and 3 the same zeros are plotted with respect to a system parameter $\lambda _1$ and a unit circle respectively.

### Parametric curves and exceptional points

In the Hermitian systems, the topological phase transition is seen when the parametric curve touches the origin. When the parametric encloses the origin, it corresponds to topological phase^50^. Similarly, in the case of non-Hermitian systems, the topological phase is represented when the parametric curves enclose the EP whereas the transition is marked when it touches EP when the system is going from one topological phase to another. At EP the eigenstates of corresponding complex eigenvalues coalesce becoming degenerate and also at these points the Hamiltonian is nondiagonalizable^17,55^. These EP are special kind of degenerate points at which the excitation energy becomes zero, i.e., at $h_{x}^2 + h_{y}^2 = 0$. The positions of EP are given as,

8 $$\begin{aligned} h_{xr} = - h_{yi},\;\;\; \text {and} \;\;\; h_{yr} = h_{xi} \text {or} h_{xr} = h_{yi},\;\;\; \text {and} \;\;\; h_{yr} = -h_{xi}. \end{aligned}$$

Where $h_{yr}$, $h_{yi}$, $h_{xr}$ and $h_{xi}$ are the real and imaginary parts of components $h_{y}$ and $h_{x}$ of the model Hamiltonian (The detailed derivation is relegated to the ''Method'' Section). Depending on the value of the non-Hermitian factor $\gamma$, the positions of the EP change along the $\chi _{y}$ axis.

**Figure 4:**
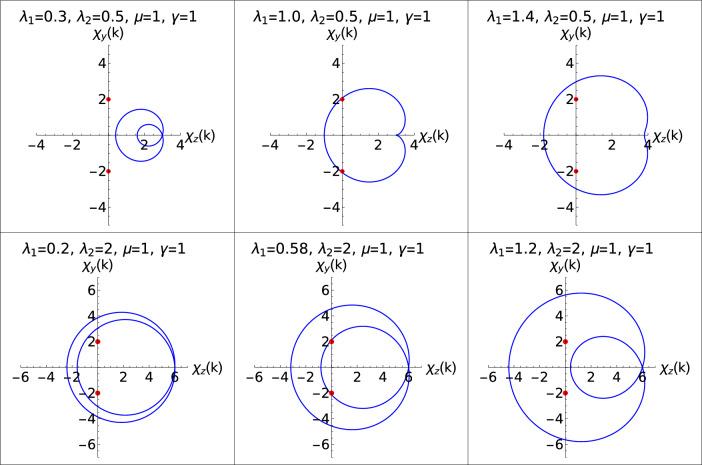
Parametric plots are represent the topological phase transitions in the positive $\lambda _2$ region. Upper and lower panel corresponds to W=1 and $W=2$ to 1 respectively. Middle plot in both the panels represents the parametric curves at critical points.

The parametric curves for topological phase transitions, W=0–1 and $W=2$ to 1 are studied in the Fig. 4. Two red spots on the $\chi _{y}$ axis at 2,-2 shown in the Fig. 4 are the positions of EP through which the transition occurs. In the positive $\lambda _2$ region, the critical lines remain linear in presence of the non-Hermiticity. We observe a parallel shift in the critical lines for increase in the value of $\gamma$. Positive $\lambda _2$ region offers two topological phase transition, W=0–1 and W=2 to 1.

In the upper panel of the Fig. 4, gapped phase $W=0$ (left plot), transition point (middle plot) and gapped phase $W=1$ (right plot) are shown.

**Figure 5:**
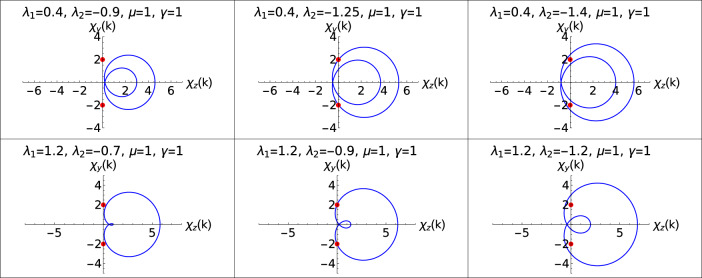
Parametric plots representing the topological phase transitions in the negative $\lambda _2$ region. Upper and lower panel corresponds to W=0–1 for different values of $\lambda _1$ respectively. Middle plot in both the panels represents the parametric curves at critical points.

**Figure 6:**
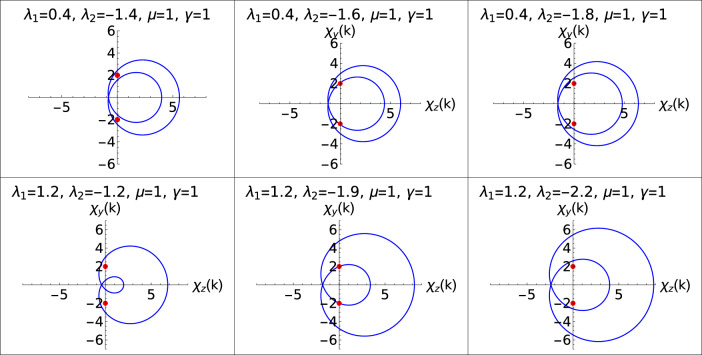
Parametric plots representing the topological phase transitions in the negative $\lambda _2$ region. Upper and lower panel corresponds to W=1–2 for different values of $\lambda _1$ respectively. Middle plot in both the panels represents the parametric curves at critical points.

Similarly in the lower panel, transition from $W=2$ to 1 is depicted. As seen from the phase diagram, Fig. 1, critical lines in the negative $\lambda _2$ region behave in a different manner where it is no longer linear or follows a parallel shift when compared to the critical lines in positive $\lambda _2$ region. In order to understand the topological phase transitions in the negative $\lambda _2$ region, the parametric curves are plotted to depict the transition between gapped phases as shown in the Figs. 5 and 6.

The deflection of the critical line “qr” as shown in the phase diagram Fig. 1 is a novel behavior observed in the non-Hermitian system. Figure 5 shows the transition between W=0–1. Similarly, parametric plot representing W=1–2 for two different values of $\lambda _1$ is also shown in the Fig. 6.

In the Fig. 6, we present the topological phase transition between $W=1$ and 2 for two different values of $\lambda _1$. Upper panel is for $\lambda _1 = 0.4$ where left plot corresponds to $W=1$ phase, middle plot corresponds the transition and the right plot corresponds to topological phase $W=2$.

Parametric curves show, gap closing at the topological phase transition points for two different values of $\lambda _1$. Deviated critical lines shown in the phase diagram, Fig. 1, is supported by the parametric curves. Due to this deviation of critical line, the multicritical point is displaced and as a result, the nature of the multicrititcal point is also changed. Observing Figs. 4, 5 and 6, they clearly show that the parametric curve encloses the EP which corresponds to topologically non-trivial phase and similarly it does not enclose the EP which corresponds to topologically trivial phase. With Changed nature of criticality also the parametric curves presents a simple form to characterize the topological phases.

## Bulk edge properties at quantum criticality

In this section, we study the concept of bulk edge properties for the model in presence of $\gamma$. In the topological systems, the concept of bulk-boundary correspondence holds together the bulk and edge properties which establishes a robust relation between them. In Hermitian case, the validation of bulk and edge properties for the gapped phases is well established^56^, and since the MZMs appear at the criticality too, the validation of bulk-boundary correspondence at criticality is also observed^47^. The scenario of bulk-boundary correspondence in non-Hermitian case entirely different when compared to the Hermitian case. Although the breakdown of conventional bulk-boundary correspondence in the non-Hermitian systems has been reported^57,58^, there has been attempt to build a generalized bulk-boundary correspondence in the recent years^59–62^. We study both, edge property i.e., the robustness of MZMs at criticality and bulk property i.e., the effect of $\gamma$ on topological phases.

### Majorana zero modes at criticality

Here in this section we study the effect of non-Hermiticity $\gamma$ over the criticalities and MZMs present at the criticality. For the non-Hermitian model Hamiltonian considered, MZMs are present in their respective topological phases just as in the case of Hermitian systems. Authors of Ref.^47^ have studied the characterization and MZMs at criticality and similarly in the non-Hermitian systems, we observe the MZMs at criticality. Criticality hosts MZMs when it separates topological phases with winding number $W>0$. For example, critical lines separating $W=1$ and $W=2$ and higher order winding number hosts MZMs which is dependent on the difference between the winding number of two topological phases.

**Figure 7:**
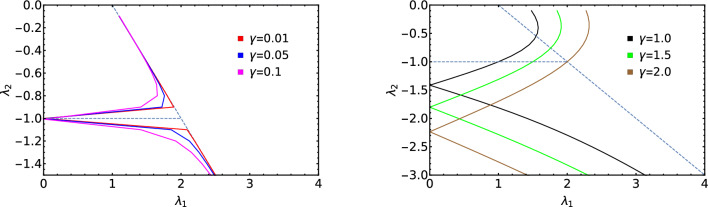
Phase diagram of $\lambda _2$ with $\lambda _1$. All the intersecting points on the negative $\lambda _2$ axis in the right plot are the positions of multicritical point for different values of $\gamma$.

**Figure 8:**
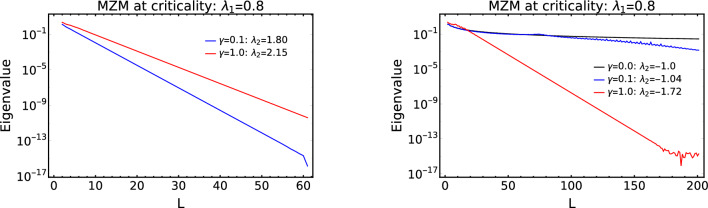
Behavior of zero mode eigenvalues at criticality “qu” and “rs” (refer to the Fig. 1) with system size for different values of $\gamma$.

In the non-Hermitian system under consideration, critical lines, “qu” and “rs”, shown in the phase diagram, Fig. 1, hosts the MZMs because they separate topological phases $W=2$ and $W=1$. In the Hermitian counterpart of the model Hamiltonian, it has three critical lines where one of them is present in the negative $\lambda _2$ region which separates $W=0$ and $W=2$ topological phases. But with the introduction of the non-Hermitian factor $\gamma$, the the critical line that separates $W=0$ and 2 topological phases no longer exist which can be seen from the Fig. 7. The phase between the critical line “qu” and “rs” is acquired by a topological phase $W=1$. Hence with the introduction of $\gamma$ induces a successive topological phase transition between the $W=0$, $W=1$ and $W=2$. Whereas in the Hermitian case $(\gamma =0)$ it was only a topological phase transitions between $W=0$ and $W=2$. Due to the appearance of topological phase $W=1$ in between the critical lines “qu” and “rs”, the critical line “rs” hosts MZMs at criticality.

The acquirement of the topological phase $W=1$ can be clearly seen from the Fig. 7 where for lesser $\gamma$ values which is represented in the left plot, the acquired area is less and as the value of $\gamma$ is increased the acquired area is more as seen from the right plot.

Since the critical lines “qu” and “rs” host MZMs, the stability of these MZMs are studied in the Fig. 8. For the critical line “qu”, the zero mode eigenvalues decay exponentially for different values of $\gamma$. For the critical line “rs”, the situation is a bit different because if $\gamma$ tends to zero, the critical line of the Hermitian Hamiltonian does not host the MZMs at criticality which can be see from the right plot (black line) of Fig. 8. As the values of $\gamma$, the critical line “rs” hosts MZMs because for finite $\gamma$ value it separates $W=1$ and $W=2$ topological phases. Hence the MZMs localize at the criticality “rs” for larger system size when compared to the localization of MZMs at the criticality “qu” which can be seen from the Fig. 8.

With higher values of $\gamma$, the MZMs on the critical line “rs” becomes more robust because the acquired area by the topological phase $W=1$ is more. The acquirement by the topological phase $W=1$ is caused by the displacement of the multicritical point from its previous position $\lambda _2=-1$ and $\lambda _1= 2.0$ (Hermitian case) to its new position $\lambda _2=\sqrt{\mu ^2 + \gamma ^2}$ and $\lambda _1 = 0$ (non-Hermitian case).

### Effect of non-Hermiticity on the topological phases and criticality

Here in this section we extend the study of effect of non-Hermiticity $\gamma$ over the criticalities using the quantity which measures the variation of the difference of the critical lines with changing $\gamma$.

In the Hermitian case, extended Kitaev chain possess three gapped topological phases and respectively three critical lines. The additional feature of the Hermitian extended Kitaev chain is the presence of multicritical point at $\lambda _1 = 2$ and $\lambda _2 = -1$ which has been extensively studied in reference^47,48^. From the ZMS the new critical lines were identified and the phase diagram is constructed (Fig. 1). As presented in the Fig. 7, the positions of the critical lines change for different values of $\gamma$.

**Figure 9:**
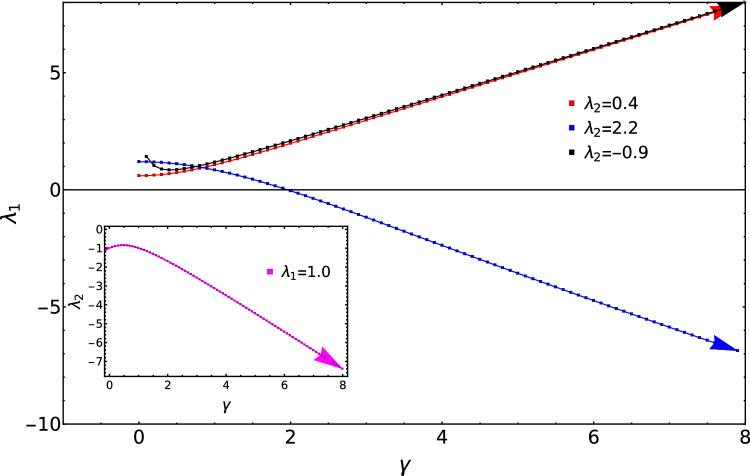
Behavior of critical lines with $\gamma$ for different values of $\lambda _1$ and $\lambda _2$. Inset represents the behavior of critical line with $\lambda _2$.

Considering topological phase transition between all the topological phases, the critical lines move as shown in the Fig. 9. In the Fig. 9, we present the movement of critical lines with respect to $\gamma$ for fixed values of $\lambda _1$ and $\lambda _2$.

Red, black and blue curves of Fig. 9 are respectively for $\lambda _2 = 0.4, 2.2$ and $-0.9$ representing topological phase transitions $w = 0$ to 1, $W=1$ to 2 and $W=0$ to 1. It reveals from this study that for $W=0$ to 1 transition in the negative $\lambda _2$ region the critical points does not continuously increase with respect to $\gamma$ like the red curve, instead they decrease initially and then increase with respect to the increase in the value of $\gamma$.

This happens because, critical point for $\lambda _2 = -0.9$ move backwards first and then moves forward towards higher values of $\lambda _1$. For $W=0$ to 1 and $W=2$ to 1 topological phase transitions, the backward movement of the critical line is not observed.

The arrow mark shows the direction of the movement of the critical lines. Blue curve corresponding to $W=2$ to 1 decreases and moves eventually to the negative $\lambda _1$ plain because, as the value of $\gamma$ is increased, the critical line for $\lambda _2 = 2.2$ moves backward and eventually goes to negative $\lambda _1$ plain because the same critical line is continued in the negative $\lambda _1$ plain. Both red and blue curves show increase and decrease respectively unlike the black curve.

Inset of the Fig. 9 corresponds to the critical lines plotted with respect to $\gamma$ for fixed value of $\lambda _1 = 1.0$. This represent the transition from $W=1$ to 2 topological phases in negative $\lambda _2$ regime. Even in this phase transition, there is a slight increase in the values of critical points and as the value of $\gamma$ is increased, the value of the critical points decrease continuously. This is also because of the backward movement for certain value of $\gamma$ and the forward movement for the rest of the values of $\gamma$.

**Figure 10:**
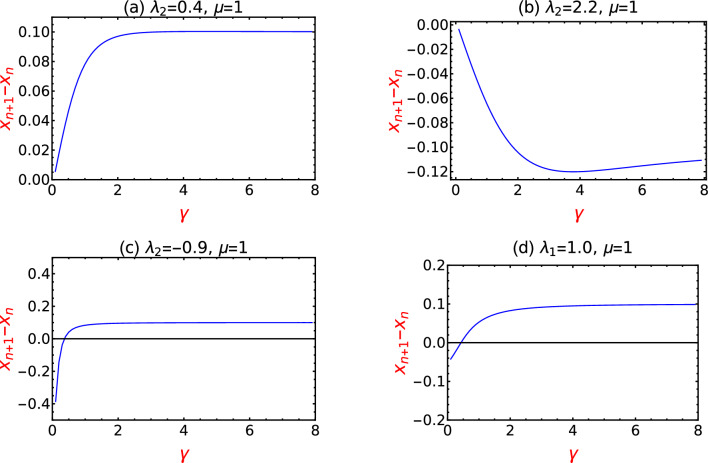
Variations in the difference of critical lines $(x_{n+1} - x_{n})$ with $\gamma$ for fixed values of $\lambda _1$ and $\lambda _2$.

Studying the movement and the direction of the critical points with respect to $\gamma$ for all the transitions, the rate of receding in both positive and negative direction with respect to $\gamma$ is also studied which is presented in the Fig. 10.

The difference between the values of the critical points represented as $x_{n+1} - x_{n}$ is calculated and it is plotted with respect to the $\gamma$. The quantity $x_{n+1} - x_{n}$ corresponds to the difference of the critical lines with respect to different values of $\gamma$ starting from $\gamma =1$ to 8. The quantity $x_{n+1} - x_{n}$ signifying the rate of receding of critical points with respect to $\gamma$ reveals some interesting behavior in the Fig. 10. Plots (a) and (b) of the Fig. 10 in the upper panel corresponds to the values of $\lambda _2 = 0.4$ and 2.2. These values represent the transition from $W=0$ to 1 and $W=2$ to 1 topological phases.

In the plot (a) of the Fig.10, the difference, $x_{n+1} - x_{n}$ rises sharply and saturates at 1 signifying that spacing between the critical points increases rapidly till $\gamma = 2$ and for $\gamma > 2$, the spacing remains constant which is depicted by the saturation of the curve. The quantity, $x_{n+1} - x_{n}$ is negative in the plot (b) signifying only the backward movement of critical points with respect to $\gamma$ and it does not mean that spacing between the critical points are negative.

Plot (b) of Fig. 10 corresponds to $W=2$ to 1 topological phase transition and as the plot shows, the difference becomes large with increasing $\gamma$ value but in the opposite direction. Critical points show an interesting behavior in the negative quadrant of $\lambda _2$ (plot (c) and (d) of Fig. 10) where they show both and forward movement with respect to increase in the values of $\gamma$. Due to the modification of critical lines where they are no longer linear and also with he displacement of multicritical point, they show the corresponding behavior (refer Fig. 10) for increasing values of $\gamma$. This analysis shows a detailed effects of $\gamma$ on the topological phases and criticality.

## Discussion

We have computed the critical lines of non-Hermitian extended Kitaev chain by using an elegant method of zero mode solutions. We have investigated and observed an interesting phenomenon where the critical lines are modified. It also shows the disappearance of multicritical point from its usual location. Parametric curve study has been performed in order to validate the nature of the critical lines. The topological characterization studied using parametric curves results in calculating the EP and its dependence on the non-Hermitian factor. We also have investigated the MZMs at criticality and its stability. We have observed a displacement in the multicritical point position due to non-Hermitian factor $\gamma$. Finally the effect of $\gamma$ on the topological phases has studied by calculating the difference of the positions of critical lines for different values of $\gamma$. This shows the rate at which the critical lines recede. Critical points for corresponding values of $\gamma$ have been calculated and its difference has studied. This showed the rate of receding which gives the dynamics of critical lines for increasing values of $\gamma$.

## Methods

### Zero mode solutions

The model Hamiltonian can be written as,

9 $$\begin{aligned} H_k = -(\chi _{z}(k) \sigma _z + \chi _{y}(k) \sigma _y), \end{aligned}$$

where $$\chi _{z} (k) = 2 \lambda _1 \cos k + 2 \lambda _2 \cos 2k - 2(\mu + i \gamma ),$$ and $$\chi _{y} (k) = 2 \lambda _1 \sin k + 2 \lambda _2 \sin 2k.$$

Substituting the exponential forms of $\cos k$ and $\sin k$, Eq. (9) becomes,

10 $$\begin{aligned} H_k= & {}  2 \lambda _1 \frac{1}{2} (e^{-ik} + e^{ik}) + 2 \lambda _2 \frac{1}{2} (e^{-2ik} + e^{2ik} - 2 (\mu +i \gamma )) \sigma _z \nonumber + i 2 \lambda _1 \frac{1}{2} (e^{ik} - e^{-ik}) + 2 \lambda _2 \frac{1}{2}(e^{2ik} - e^{-2ik})  \sigma _y. \end{aligned}$$

We replace $e^{-ik} = e^{q}$, Eq. (10) becomes,

11 $$\begin{aligned} H_q= & {} 2 \lambda _1 \frac{1}{2} (e^{q} + e^{-q}) + 2 \lambda _2 \frac{1}{2} (e^{2q} + e^{-2q}) - 2 (\mu +i \gamma ) \sigma _z \nonumber + i2 \lambda _1 \frac{1}{2} (e^{-q} - e^{q}) + 2 \lambda _2 \frac{1}{2}(e^{-2q} - e^{2q}) \sigma _y. \end{aligned}$$

We make $H^2_q=0 \;$^31^, to obtain the zero solutions for certain $q$ where $\sigma_z$ and $\sigma_y$ square to 1 or become 0 due to anticommutation.

12 $$\begin{aligned} 2 \lambda _1 \frac{1}{2} (e^{q}+e^{-q}) + 2 \lambda _2 \frac{1}{2} (e^{2q}+e^{-2q} - 2 (\mu +i \gamma )) + 2 \lambda _1 \frac{1}{2} (e^{-q}-e^{q}) + 2 \lambda _2 \frac{1}{2} (e^{-2q}-e^{2q}) = 0 \end{aligned}$$

Simplifying the Eq. (12), we end up with a quadratic equation,

13 $$\begin{aligned} 2 \lambda _1 \frac{1}{2} e^{q} + 2 \lambda _2 \frac{1}{2} e^{2q} + 2 (\mu +i \gamma ) + 2 \lambda _1 \frac{1}{2} e^{q} + 2 \lambda _2 \frac{1}{2} e^{2q} = 0. \end{aligned}$$

Simplifying the Eq. (13) to a quadratic form and substituting $e^q = X$,

14 $$\begin{aligned} \lambda _2 X^2 + \lambda _1 X - (\mu +i \gamma ) = 0. \end{aligned}$$

The roots of this quadratic Equation is given by,

15 $$\begin{aligned} X = \frac{-\lambda _1 \pm \sqrt{\lambda _1^2 + 4 \lambda _2 (\mu +i \gamma )}}{2 \lambda _2} \end{aligned}$$

### Exceptional points

Exceptional points arise naturally in the non-Hermitian systems because of the complex nature of the system. In the Hermitian systems, the singularity or the critical points are obtained in a straight forward manner. In non-Hermitian systems, specially in the *PT* symmetric Hamiltonians, obtaining gap closing points or the so called exceptional points is a bit tricky.

For example, considering a 2$\times$2 Hamiltonian,

16 $$\begin{aligned} \left( \begin{matrix} i \gamma &{}&{} -J\\ -J &{}&{} - i \gamma \end{matrix} \right) , \end{aligned}$$

where *J* and $\gamma$ are non negative numbers.

The energy eigenvalues are given by,

17 $$\begin{aligned} E = \pm \sqrt{J^2 - \gamma ^2}. \end{aligned}$$

From the Eq. (19), one can see that the eigenvalues are real when $J > \gamma$ which marks the *PT* unbroken region. For $J<\gamma$, the eigenvalues are all complex which marks the *PT* broken region. For $J = \gamma$ is the transition point between the *PT* unbroken and broken phases.

Since exceptional points arises naturally irrespective of systems obeying *PT* symmetry, model Hamiltonian under consideration also possess exceptional points.

Exceptional points for such systems can be seen from an example.

Consider a 2$$\times$$2 Hamiltonian,

18 $$\begin{aligned} \left( \begin{matrix} h_z(k)+i\gamma &{}&{} h_y(k)\\ -h_y(k) &{}&{} - (h_z(k)+i\gamma ) \end{matrix} \right) , \end{aligned}$$

where $h_y$ and $h_z$ are the components of the Hamiltonian.

The eigenvalues are given by,

19 $$\begin{aligned} E = \pm \sqrt{(h_y)^2 + (h_z + i\gamma )^2}. \end{aligned}$$

To find the exceptional points is nothing but finding the condition when $| E| = 0$. Hence, |*E*| will become zero, when $h_z = 0$ and $h_y = \pm \gamma$. The condition $h_y = \pm \gamma$ gives the location of the exceptional points.
