# Author Correction: Topological quantum criticality in non-Hermitian extended Kitaev chain

**DOI:** 10.1038/s41598-023-49938-w

**Published:** 2024-01-05

**Authors:** S. Rahul, Sujit Sarkar

**Affiliations:** 1 Department of Theoretical Sciences, Poornaprajna Institute of Scientific Research, 4, Sadashivanagar, Bangalore, 560080 India; 2 Department of Theoretical Sciences, Poornaprajna Institute of Scientific Research, Bidalur Post, Devanhalli, Bangalore Rural, 562110 India; 3 Graduate Studies, Manipal Academy of Higher Education, Madhava Nagar, Manipal, 576104 India; 4 Department of Physics, IIT Madras, Chennai, India

Correction to: *Scientific Reports* 10.1038/s41598-022-11126-7, published online 28 April 2022

The original version of this Article contained a repeated error, where the sign “*CP*” was incorrectly given as “*Cq*”. As a result of this error, in the Results,

“The Hermitian model consists of three critical lines (solid black line) “AB”, “BD” and “Cq” distinguishing topological phases W = 0, W = 1 and W = 2.”

now reads:

“The Hermitian model consists of three critical lines (solid black line) “AB”, “BD” and “CP” distinguishing topological phases W = 0, W = 1 and W = 2.”

And, in the Results section, under the subheading ‘Zero mode analysis for topological characterization’,

“From the Fig. 1, it can be clearly observed that the critical line “Cq” is not present for the non-Hermitian case (critical lines presented in red color).”

now reads:

“From the Fig. 1, it can be clearly observed that the critical line “CP” is not present for the non-Hermitian case (critical lines presented in red color).”

Additionally, the original version of this Article contained errors in the sign in the zero mode solutions in the Method section, where

**Zero mode solutions**.

The model Hamiltonian can be written as,

9 $$\begin{aligned} H_k = \chi _{z}(k) \sigma _z + \chi _{y}(k) \sigma _y, \end{aligned}$$

where $$\chi _{z} (k) = 2 \lambda _1 \cos k + 2 \lambda _2 \cos 2k - 2(\mu + i \gamma ),$$ and $$\chi _{y} (k) = 2 \lambda _1 \sin k + 2 \lambda _2 \sin 2k.$$

Substituting the exponential forms of $\cos k$ and $\sin k$, Eq. (9) becomes,

10 $$\begin{aligned} H= & {} \left[ 2 \lambda _1 \frac{1}{2} (e^{-ik} + e^{ik}) + 2 \lambda _2 \frac{1}{2} (e^{-2ik} + e^{2ik} + 2 (\mu +i \gamma ))\right] \sigma _z \nonumber \\&+ i \left[ 2 \lambda _1 \frac{1}{2} (e^{ik} - e^{-ik}) + 2 \lambda _2 \frac{1}{2}(e^{2ik} - e^{-2ik}) \right] \sigma _y. \end{aligned}$$

We replace $e^{-ik} = e^{q}$, Eq. (10) becomes,

11 $$\begin{aligned} H= & {} \left[ 2 \lambda _1 \frac{1}{2} (e^{q} + e^{-q}) + 2 \lambda _2 \frac{1}{2} (e^{2q} + e^{-2q}) + 2 (\mu +i \gamma )\right] \sigma _z \nonumber \\&+ i \left[ 2 \lambda _1 \frac{1}{2} (e^{-q} - e^{q}) + 2 \lambda _2 \frac{1}{2}(e^{-2q} - e^{2q}) \right] \sigma _y. \end{aligned}$$

To find the zero mode solutions, we make $H^2 = 0$. By solving the Eq. (11), we get,

12 $$\begin{aligned} 2 \lambda _1 \cosh q + 2 \lambda _2 \cosh 2q - 2(\mu +i \gamma ) = \pm 2 \lambda _1 \sinh q + 2 \lambda _2 \sinh 2q. \end{aligned}$$

Equation 12 shows that there will be more than one solution. Considering the Eq. (11) and squaring both sides with $H=0$, we get,

13 $$\begin{aligned} \left( 2 \lambda _1 \cosh q + 2 \lambda _2 \cosh 2q - 2(\mu +i \gamma )\right) + i \left( 2 \lambda _1 \sinh q + 2 \lambda _2 \sinh 2q\right) = 0. \end{aligned}$$

Substituting back the exponential forms to the respective terms, we get,

14 $$\begin{aligned}&\left[ 2 \lambda _1 \frac{1}{2} (e^{q} + e^{-q}) + 2 \lambda _2 \frac{1}{2} (e^{2q} + e^{-2q} + 2 (\mu +i \gamma ))\right] \nonumber \\&\quad + \left[ 2 \lambda _1 \frac{1}{2} (e^{-q} - e^{q}) + 2 \lambda _2 \frac{1}{2}(e^{-2q} - e^{2q}) \right] = 0 \end{aligned}$$

Simplifying the Eq. (14), we end up with a quadratic equation,

15 $$\begin{aligned} 2 \lambda _1 \frac{1}{2} e^{q} + 2 \lambda _2 \frac{1}{2} e^{2q} + 2 (\mu +i \gamma ) + 2 \lambda _1 \frac{1}{2} e^{q} + 2 \lambda _2 \frac{1}{2} e^{2q} = 0. \end{aligned}$$

Simplifying the Eq. (15) to a quadratic form and substituting $e^q = X$,

16 $$\begin{aligned} \lambda _2 X^2 + \lambda _1 X + (\mu +i \gamma ) = 0. \end{aligned}$$

The roots of this quadratic Equation is given by,

17 $$\begin{aligned} X = \frac{-\lambda _1 \pm \sqrt{\lambda _1^2 + 4 \lambda _2 (\mu +i \gamma )}}{2 \lambda _2} \end{aligned}$$

The roots Eq. (17) are the solutions of zero modes.

now reads:

**Zero mode solutions**.

The model Hamiltonian can be written as,

1 $$\begin{aligned} H_k = -\left( \chi _{z}(k) \sigma _z + \chi _{y}(k) \sigma _y\right) , \end{aligned}$$

where $$\chi _{z} (k) = 2 \lambda _1 \cos k + 2 \lambda _2 \cos 2k - 2(\mu + i \gamma ),$$ and $$\chi _{y} (k) = 2 \lambda _1 \sin k + 2 \lambda _2 \sin 2k.$$

Substituting the exponential forms of $\cos k$ and $\sin k$, Eq. 1 becomes,

2 $$\begin{aligned} H_k= & {} 2 \lambda _1 \frac{1}{2} (e^{-ik} + e^{ik}) + 2 \lambda _2 \frac{1}{2} (e^{-2ik} + e^{2ik} + 2 (\mu +i \gamma ))\sigma _z\nonumber \\{} & {} + i 2\lambda _1 \frac{1}{2} (e^{ik} - e^{-ik}) + 2 \lambda _2 \frac{1}{2}(e^{2ik} - e^{-2ik}) \sigma _y. \end{aligned}$$

We replace $e^{-ik} = e^{q}$, Eq. 2 becomes,

3 $$\begin{aligned} H_q= & {} 2 \lambda _1 \frac{1}{2} (e^{q} + e^{-q}) + 2 \lambda _2 \frac{1}{2} (e^{2q} + e^{-2q}) + 2 (\mu +i \gamma ) \sigma _z\nonumber \\{} & {} + i 2 \lambda _1 \frac{1}{2} (e^{-q} - e^{q}) + 2 \lambda _2 \frac{1}{2}(e^{-2q} - e^{2q}) \sigma _y. \end{aligned}$$

We make $H_q^2 = 0$^31^, to obtain the zero solutions for certain *q* where $\sigma _z$ and $\sigma _y$ square to 1 or become 0 due to anticommutation.

4 $$\begin{aligned} 2 \lambda _1 \frac{1}{2} (e^{q} + e^{-q}) + 2 \lambda _2 \frac{1}{2} (e^{2q} + e^{-2q} + 2 (\mu +i \gamma )) + 2 \lambda _1 \frac{1}{2} (e^{-q} - e^{q}) + 2 \lambda _2 \frac{1}{2}(e^{-2q} - e^{2q}) = 0 \end{aligned}$$

Simplifying the Eq. 4, we end up with a quadratic equation,

5 $$\begin{aligned} 2 \lambda _1 \frac{1}{2} e^{q} + 2 \lambda _2 \frac{1}{2} e^{2q} + 2 (\mu +i \gamma ) + 2 \lambda _1 \frac{1}{2} e^{q} + 2 \lambda _2 \frac{1}{2} e^{2q} = 0. \end{aligned}$$

Simplifying the Eq. 5 to a quadratic form and substituting $e^q = X$,

6 $$\begin{aligned} \lambda _2 X^2 + \lambda _1 X + (\mu +i \gamma ) = 0. \end{aligned}$$

The roots of this quadratic Equation is given by,

7 $$\begin{aligned} X = \frac{-\lambda _1 \pm \sqrt{\lambda _1^2 + 4 \lambda _2 (\mu +i \gamma )}}{2 \lambda _2} \end{aligned}$$

Finally, the original version of this Article contained an error in the legend of Fig. 2.

“Zero mode solutions plotted with respect to the parameter λ_1_ shows both W = 0 to W = 1(λ_2_ = 0.5) and W = 1 to W = 2(λ_2_ = 2.0) topological phase transitions. The red dot (p1 and p2) represents the transition points. The ZMS, X_+_ (red) and X_−_ (blue) are plotted in y-axis.”

now reads:

“Zero mode solutions plotted with respect to the parameter λ_1_ shows both W = 0 to W = 1(λ_2_ = 0.5) and W = 1 to W = 2(λ_2_ = 2.0) topological phase transitions. Blue dots (p1 and p2) represent the transition points. The ZMS, X_+_ (red) and X_−_ (blue) are plotted in y-axis.”

The original Article has been corrected.

